# Application of Induced Pluripotent Stem Cell Technology to the Study of Hematological Diseases

**DOI:** 10.3390/cells6010007

**Published:** 2017-03-08

**Authors:** Mailin Li, Pasquale Cascino, Simone Ummarino, Annalisa Di Ruscio

**Affiliations:** Department of Translational Medicine, University of Eastern Piedmont, Novara 28100, Italy; mailin.li@med.uniupo.it (M.L.); pasquale.cascino@med.uniupo.it (P.C.); simone.ummarino@uniupo.it (S.U.)

**Keywords:** regenerative medicine, blood diseases, reprogramming, cancer

## Abstract

The burst of reprogramming technology in recent years has revolutionized the field of stem cell biology, offering new opportunities for personalized, regenerative therapies. The direct reprogramming of somatic cells to induced pluripotent stem cells (iPSCs) has provided an invaluable tool to study and model a wide range of human diseases. Here, we review the transforming potential of such a strategy in research and in therapies applicable to the hematology field.

## 1. Introduction

### 1.1. Stem Cells: Features

Stem cells are rare, undifferentiated cells in an organism and are defined by their properties of (1) self-renewal, the ability to undergo numerous cycles of cell division while maintaining an undifferentiated state; and (2) potency, the ability to generate cells of many lineages. Stem cells function in early development and in adult organisms to maintain and repair tissue integrity. However, their number and potency diminish with age. 

**Figure 1:**
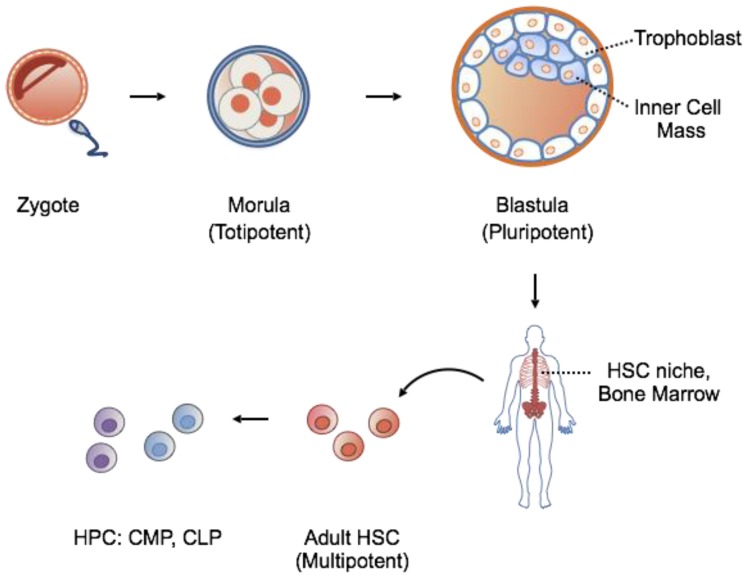
The hierarchy of stem cell potency during embryonic to adult development. In general, increasing cell specialization parallels a decrease in potency. HSC, hematopoietic stem cell; HPC, hematopoietic progenitor cell; CMP, common myeloid progenitor; CLP, common lymphoid progenitor.

Based on their potency, stem cells can be classified according to a hierarchical order ranging from totipotent, to pluripotent, to multipotent stem cells (Figure 1). Totipotent stem cells of the early cleavage stages are able to generate an entire organism when separated. Further cell division and blastulation gives rise to the trophoblast, eventually forming the placenta, and inner mass cells, destined to become the fetus. Isolation of inner mass cells yields pluripotent embryonic stem cells capable of generating all three embryonic germ layers: endoderm, ectoderm and mesoderm. In contrast to the high population of stem cells existing in early development, adult stem cells are rare, exist among differentiated tissues in specialized niches, and function primarily in tissue maintenance and repair. Adult stem cells are multipotent, lineage-restricted cells, and are capable of generating a single germ layer, often of a single organ system. 

The hematopoietic stem cell (HSC) is the best-studied and well-characterized multipotent stem cell. It resides in the adult bone marrow niche and is able to regenerate all the cellular components of the blood. For these reasons, HSCs represent an attractive target for regenerative medicine. 

### 1.2. Stem Cells: Source for Potential Therapy

The differentiation capacity of HSCs has been heavily utilized in regenerative medicine and other stem cell-based therapies. CD34+ HSCs can be collected from the bone marrow, umbilical cord blood, or from peripheral blood following granulocyte colony stimulating factor (G-CSF)-mobilization from the bone marrow [1,2]. Transplantation of HSCs (HSCT) has become the standard treatment for numerous hereditary diseases and malignant blood disorders [3]. The attractiveness of this approach stems from the possibility to regenerate all the cellular components of the blood system and to permanently restore a functioning immune system damaged by natural or acquired conditions. Regardless of the HSC source, problems associated with transplantation include reliance on donors, risk of infection under immunosuppressive drugs, and immunological compatibility determined by the degree of “*donor-recipient matching*” determined as the percentage of identity shared between the Human Leukocyte Antigen Loci (HLA) complex of two individuals. The matching point greatly limits the number of donor-recipient pairs, with donors found primarily among HLA-identical siblings, other family members, and from unrelated marrow donor registries [4]. Even with compatible major HLA loci, mismatches at minor HLA loci can lead to graft versus host disease (GVHD), in which successfully grafted donor T lymphocytes mount an immune response against host antigens, leading to excess inflammation and immune response. 

Alternatively, HSCs can be harvested from more primitive and “antigenically naive” umbilical cord blood donations. However, this approach is largely restrained by the number of cells available per donation; a single donation provides enough HSCs only for child bone marrow transplantations [5]. In the attempt to overcome source material limitations, numerous ex vivo expansion protocols have been tested, but with low or modest effects [6]. While some advances have been made over time using double cord blood grafts [7] and increased biobanking [5], finding an alternative source of stem cell material remains a necessity [6].

Embryonic stem cells (ESCs) are an alternative to transplantation of adult stem cells [8]. Unlike adult stem cells, ESCs survive endlessly in culture under the right conditions [9] and can be differentiated to the targeted cell-lineage, producing scalable and homogenous sources of transplantation material. However, the use of ESCs is hindered not only by limited access to and low amounts of source material, but also by ethical concerns related to working with embryonically-derived cells. 

Given the limitations associated with HSCT, one of the major tasks in the hematology field remains the production of a reliable and scalable source of efficient and complete long-term engrafting HSCs. In addition to their direct therapeutic application for transplantation, HSCs provide a source of mature hematopoietic cells for other therapeutic purposes such as red blood cell and platelet transfusions, drug screenings, and modeling of both human development and hematological malignancies.

## 2. Induced Pluripotent Stem Cells: A Novel Solution to an Old Problem

The generation of patient-derived HSCs through induced pluripotent technology led to an appealing strategy to overcome the technical and ethical barriers of working with HSCs and ESCs (Table 1). 

Before 2006, the pluripotent state was a property limited only to ESCs, and their use and application in research and clinic were impaired by ethical concerns associated with their derivation from embryonic material. The discovery by Takahashi and Yamanaka that ectopic expression of four transcription factors (OCT3/4, KLF4, SOX2 and MYC) [10] could restore adult, somatic cells to their pluripotent state, revealed the critical steps of a process named “reprogramming” through which differentiated cells can return to a stem-cell-like state. 

Hence, the advent of induced pluripotent stem cells (iPSCs) overcame many of the dilemmas of working with stem cells and generated great optimism in the field of hematology. Indeed, iPSCs have extensive self-renewal capacity, could differentiate into any cell type, and represent an unlimited source of research and therapy material. 

Specifically, the generation of iPSC-derived hematopoietic stem cells could theoretically solve all the issues connected with HLA compatibility by eliminating complications associated with transplantation, such as the use of immunosuppressive drugs, graft versus host disease, etc. Up-to-date, only a few clinical trials have tested the safety of using pluripotent stem cells for therapeutic purposes even if generation, differentiation and gene-corrected-iPSCs lineages could provide HLA-matched cell types for all pathological tissues and organs of interest, in unlimited quantities [11]. In this view iPSCs opened a new avenue of personalized therapy with the potential to develop patient-specific cells with theoretically no risk of rejection as well as providing an excellent tool to study personalized human biology. 

## 3. Generation of iPSCs

**Figure 2:**
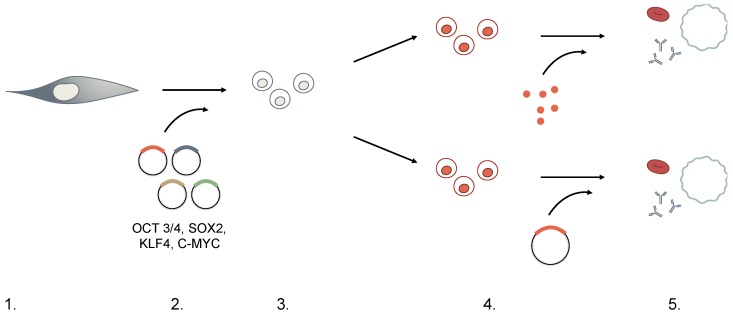
Selection stages for generation of iPSCs.

The first strategies conceived to induce pluripotency, such as somatic cell nuclear transfer (cloning) or fusion of somatic cells with embryonic stem cells (ESCs) [12] were accompanied by technical, ethical, and logistical barriers restricting the applicability of these pluripotent cells in both research and therapy. Direct generation of pluripotent cells without the use of embryonic material has emerged as a more suitable approach to facilitate functional analyses with fewer ethical implications. Cellular reprogramming can be achieved by ectopic delivery in somatic cells of four transcription factors known as OSKM (OCT3/4, SOX2, KLF4, and c-MYC, with or without Lin28) [10,13,14,15] under specific culturing conditions. While many combinations of factors have shown to successfully generate iPSCs, regardless of the factors used, current concerns of iPSCs technology focus on the method of effective delivery mediating the balance between cellular transformation versus temporary induction (Figure 2 and Table 2). To this end, several strategies have been developed to induce pluripotency:

### 3.1. Moloney-Based Retrovirus

Direct reprogramming was initially performed in mouse fibroblasts through retroviral transduction of 24 candidate genes, all implicated in the establishment and maintenance of the pluripotent state of somatic cells. Identification of these candidates led to the hypothesis that all or some of them could play pivotal roles in the maintenance of ESC identity. Takahashi et al. developed an assay in which the pluripotent state could be detected upon induction of an ESC-specific marker: the *Fbx15* gene. The authors generated ESCs with a βgeo knock-in construct (Fbx15^βgeo/βgeo^ whereby ESCs homozygous for the Fbx15^βgeo/βgeo^ were resistant to extremely high concentrations of G418 antibiotic). Somatic cells that had successfully undergone reprogramming showed reactivation of *Fbx15* and were resistant to antibiotic selection, whereas somatic cells derived from *Fbx15*^βgeo/βgeo^ mice were sensitive to a normal concentration of G418 as expected in the absence of the *Fbx15* locus. Initially, Takahashi et al. introduced all 24 candidate genes into mouse embryonic fibroblasts (MEFs) derived from *Fbx15*^βgeo/βgeo^ mice by retroviral transduction. Resistant colonies were observed for 16 days after transduction. The results obtained for the drug-resistant colonies with any single factor indicated that no single candidate gene was sufficient to activate the *Fbx15* locus. No colony-formation was observed in the absence of Oct3/4 or Klf4. Removal of Sox2 resulted in only a few colonies whereas the lack of c-Myc led to G418-resistant colonies with a flatter, non-ESC-like morphology. The remaining factors did not show to be necessary for colony formation. Overall, these results indicated that Oct3/4, Klf4, Sox2, and c-Myc were the master regulators for the generation of iPSCs. Surprisingly, the authors demonstrated that Nanog was dispensable and later the four factors were demonstrated to work across a multitude of murine cell types [16], as well as rhesus monkey [13] and human [15,17,18] cells.

Although the usage of retroviral vectors carried advantages for the initial attempts, given their self-silencing property, the transcription factor expression temporal requirements are not defined. The use of these retroviruses held three main downsides: firstly, retrovirus-generated iPSCs often maintained viral gene expression and the genomic integration increases the risk of insertional mutagenesis; [19] secondly, their infectivity is limited to dividing cells, thus restricting the range of cell types that can be reprogrammed; and lastly, increased tumor incidence in chimeric mice was observed due to transgene reactivation [20].

### 3.2. HIV-Based Lentivirus

One of the methods applied to generate pluripotent cells envisioned the use of HIV-based lentivirus vectors able to transduce non-dividing cells with high expression levels [21]. Unfortunately, these vectors are poorly silenced in the pluripotent state [22], making the constitutive versions less suitable for reprogramming. Although iPSCs made with constitutive lentiviruses have been reported [23], how differentiation proceeds during continued transgene expression remains unclear. Similarly, doxycycline (dox)-inducible lentiviral vectors have been criticized for permanent genomic integration and the high risk of insertional mutagenesis. For these reasons, great effort has been made to pursuing non-integrating approaches. 

### 3.3. Transient Transfection and Adenovirus

A valid strategy to exclude the viruses’ integration in the genome includes transient transfection and adenoviral-based technologies. In particular, adenoviral delivery has been successfully used in reprogramming mouse cells. Indeed, mouse fibroblasts and liver cells were generated by non-integrating adenoviruses, transiently expressing OCT4, SOX2, KLF4, and c-MYC. These adenoviral iPSCs (adeno-iPSCs) showed DNA demethylation features of reprogrammed cells, expressed endogenous pluripotency genes, formed teratomas, and contributed to multiple tissues, including the germ line, in chimeric mice [24]. The production of virus-free iPSCs, albeit from embryonic fibroblasts, addresses a critical safety concern for potential use of iPSCs in regenerative medicine and proves the first strong evidence that insertional mutagenesis is not required for in vitro reprogramming. The efficiency of iPSC generation, however, is substantially lower than the one observed with retroviruses, suggesting that retroviral integration facilitates iPSC generation.

### 3.4. Small Molecules

Small molecules and soluble factors can recapitulate the series of transcriptional and epigenetic changes brought about by the four transcription factors and are particularly interesting given their ease of application and the lack of permanent genome modification. In this instance, valproic acid [25] enhances reprogramming efficiency with the four OSMK factors in mouse fibroblasts; BIX01294 improves reprogramming efficiencies of OCT4, KLF4 (OK)-infected neural progenitor cells by approximately 8-fold and allows reprogramming of mouse neural progenitor cells in the absence of OCT4, although with a very low efficiency and with the presence of the other three SOX2, KLF4, c-MYC (SKM)factors [26]. It is currently unknown whether small molecules alone can recapitulate the series of transcriptional and epigenetic events resulting from ectopic expression of the OSKM master regulators.

### 3.5. Protein Transduction

Another attempt to achieve pluripotency exploits the use of transducible proteins by using pSESAME, an expression vector that facilitates the generation of transducible proteins. Both OCT4 and SOX2, two of the main regulators of pluripotency in embryonic stem cells, were genetically fused with a trans-activator of transcription (TAT) protein transduction domain that promotes cellular penetration [27]. This approach provides a powerful tool for the modulation of stem cell properties without involving genetic interference.

### 3.6. Genome Editing 

Site-specific nucleases (SSNs) are the most important genome editing research tools developed in recent years [28]. Their application to repair or introduce disease-relevant mutation in iPSCs could play a fundamental role in studying and understanding pluripotency biology. The initial zinc-finger nucleases (ZFNs) and transcription activator-like effector nucleases (TALENs) platforms for genome editing in stem cells were costly and time-consuming. Their enactment as research tools, therefore, developed comparatively slowly. However, extensive work with ZFNs and TALENs demonstrated the power of genome editing and highlighted the universal impact of these SSNs platforms. The turning point arrived in 2012 with the advent of clustered regularly interspaced short palindromic repeats (CRISPR)/CRISPR-associated (Cas) technology. It was shown that within the CRISPR type-2 systems a single protein, Cas9, could function as a SSN when associated with an engineered single guide RNA (sgRNA) that bears homology to a genetic locus of interest [29]. The sgRNA substitutes the natural Cas9-associated bacterial RNAs that normally confer target specificity for the bacterial pathogen DNA and directs Cas9 to induce a blunt Double Strand Break in any target DNA with complementarity to a 20-nucleotide-long sequence in the sgRNA. In less than four years Cas9-mediated genome editing became the platform of choice to generate SSNs and to genetically modify iPSCs. By repurposing the bacterial CRISPR/Cas9 system [30] as a SSN, the need for a simple and unified platform for genome editing together with an easy way to make iPSCs was met and resolved.

Similar to the disease-modeling approach, genome editing allows us to engineer variant alleles found to be associated with specific diseases in otherwise isogeneic cellular settings, as described in Section 5. 

## 4. iPSCs and Hematopoiesis: Are We There Yet?

iPSCs can be employed to generate lineage-specific hematopoietic cells for immunotherapy, transfusion medicine, and cell therapy. Thus far, iPSC technology has been applied to produce red blood cells [31], megakaryocytes and platelets [32], [33] B [34] and T [35] lymphocytes, myelomonocytic cells [36] and natural killer cells [37]. Protocols describing lineage-specific cell generation from ESCs or iPSCs largely depend on spontaneous differentiation within embryoid bodies cultivated in lineage-specific cytokines or cocultured with stromal cells [38,39]. Major barriers of current reprogramming technologies include low efficiency and robustness. 

### 4.1. Red Blood Cells/Platelets

Red blood cells (RBC) and platelets are commonly transfused for treatment of common hematologic disorders including anemias, thrombocytopenias, and hemophilias. The downside of repeat blood transfusion is the immunogenicity of donor blood cells and potential dangers of viral infections. Ideally, iPSCs can be used to generate universal donor RBCs generated from antigenless blood type O individuals. Additionally, red blood cells and platelets are attractive iPSC-derived end products, given their anucleate nature and thus absence of oncogenic risk. Unfortunately, transplantation of iPSC-derived blood cells suffers from major limitations concerning cell maturation and low yields. iPSC-derived RBCs suffer from inefficient enucleation, mostly expressing embryonic and fetal hemoglobin [40]. The yield of RBCs must also be vastly improved to collect the number of RBCs needed for one transfusion unit. With the emergency of novel gene-editing technologies, differentiation cultures aided by the CRISPR/Cas9 system [41] have been used to increase hemoglobin levels and RBC counts, and to generate replacement products to treat congenital diseases. 

Platelet generation suffers from poor yields in the megakaryocyte to platelet differentiation step [42]. Currently, the major limitation is reliance on stromal cells and inefficiency of megakarycyte to differentiation. Further investigation of human iPSC-derived platelet production might allow the generation of large numbers of clinically applicable platelets *ex vivo*.

### 4.2. Neutrophils and Monocytes/Macrophages 

Transfusion of granulocyte population is extremely beneficial in neutropenic patients following myeloablative therapy or genetic causes [43]. However, granulocytes are only rarely used, due to (1) their short half-life and (2) difficulties in procurement and storage. Primary human monocytes/macrophages have limited proliferation potential and are difficult to transfect. Circulating monocytes are heterogenous and vary in size, granularity, morphology, and protein expression profiles to the extent that they have been classified in several subsets [44]. Therefore, development of protocols using iPSCs to allow prolonged and large-scale production of both cell types is of large interest [36]. Although in vitro-derived macrophages exhibit functional and morphological similarities to patient-derived cells, their in vivo functions remain to be evaluated.

### 4.3. Dendritic cells and Natural Killer (NK) Cells

Dendritic cells derived from xeno-free human iPSCs have been shown to be fully functional [45]. However, these cells are still awaiting in vivo testing for efficacy and procedural safety.

NKs are lymphocytes capable of killing cells with missing self (that is, HLA class I expression). NK normal laboratory cell lines are currently unavailable, and those used for research purposes derive from NK leukemia or lymphoma patients, thereby lacking important features of normal NKs. Generation of iPSCs-derived NK cells does have implications in both cancer therapy and infectious diseases [46,47]. However, the reliance on two steps of stroma coculture and the need for sorting CD34+CD45+ cells that are extremely rare in the peripheral blood hinder clinical scale-up of the protocol.

### 4.4. T and B Lymphocytes

iPSC can also be used to generate antigen specific sources of T lymphocytes for immunotherapy applications [35,48,49]. Autologous polyclonal lymphocytes can be transfected with a chimeric antigen receptor, achieving large doses of functional, antigen-specific autologous effector T lymphocytes, avoiding off-target [50]. T cells-derived iPSCs have been generated through the OP9 coculturing system or by intra-teratoma formation [51]. Few reports have demonstrated the possibility to generate B cells-derived iPSCs by intra-teratoma formation or by coculture with OP9 [34]. Remarkably, this in vivo differentiation approach led to the generation of B cells able to produce human immunoglobulin [51], suggesting the potential application of teratoma based-platforms for patient-specific customized therapies. 

### 4.5. Hematopoietic Stem Cells

In addition to generation of differentiated blood products, generation of iPSC-derived hematopoietic stem cells has been achieved through teratoma formation [51]. Teratomas provide bone marrow like niches supporting hematopoiesis and CD34+CD45+ population of progenitor cells. Isolated progenitor cells are not carcinogenic, as they are unable to undergo serial transplantation in nude mice. Amabile et al. showed that teratomas develop bone marrow-like structures that ultimately allow physiologic differentiation of HSCs as well as CD34+CD45+ progenitors cells. The iPSC-derived hematopoiesis can be augmented by co-injection with OP9 stromal cells. Intriguingly, co-injection of OP9 cells ectopically expressing delta-like 1 factor are able to sustain specific populations of hematopoietic cells in vivo (i.e., T cells), suggesting a role of the supporting stromal cells in the differentiation process [51].

## 5. iPSCs Hematological Disorders

**Figure 3:**
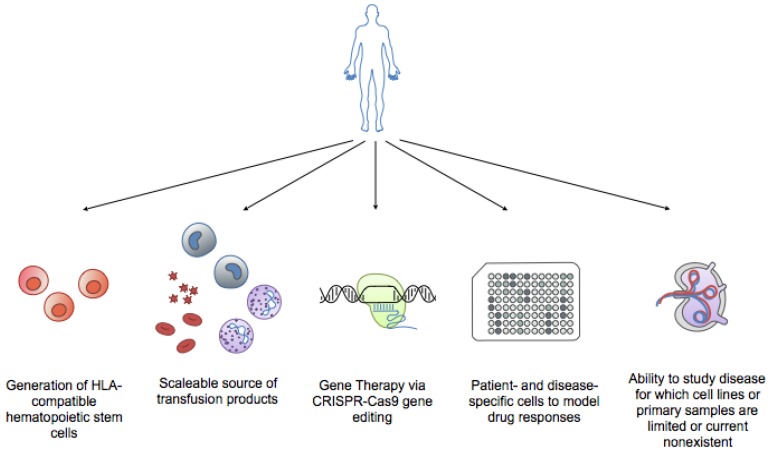
Benefits of iPSCs technology in hematology. Applications of iPSCs include generation of HLA-compatible hematopoietic stem cells for transplantation, providing a scalable source of transfusion products (i.e., red blood cells, neutrophils for neutropenias, platelets for clotting disorders), gene therapy via CRISPR/Cas9 gene editing of engineered cells (i.e., sickle cell disease, thalassemias), modeling patient and disease-specific responses to drugs, and the ability to study diseases for which cell lines or primary patient samples are limited or currently non-existent (i.e., lymphomas).

In addition to their application in transfusion and regenerative medicine, iPSCs have been used to study the pathogenesis of inherited genetic diseases [52]. Recently, it was reported that iPSCs could be generated not only from normal tissue cells but also from malignant cells [53,54]. Patient-derived iPSCs can help in elucidating the molecular mechanisms of various rare terminal diseases, including hematologic malignancies, through the establishment of in vitro cellular models as well as the development of novel targeted therapies (Figure 3).

### 5.1. iPSCs in Congenital Hematopoietic Diseases

Inherited bone marrow failure (BMF) syndromes are a group of heterogeneous disorders characterized by congenital BMF leading to single or multiple lineage cytopenias and associated with risk of developing solid organ cancers [55]. Inherited BMF syndromes often develop as a result of specific genetic mutations or polymorphism in hematopoietic stem cells which make iPSCs-based disease modeling a therapeutic tool for gene-correction approaches. At present, the only available therapy for BMF syndromes is allogenic HSC transplantation.

#### 5.1.1. Fanconia Anemia

Fanconi anemia (FA) is characterized by physical abnormalities, bone marrow failure (BMF), and increased risk for malignancy [56]. Progressive bone marrow failure with cytopenia typically occurs in the first decade, often initiated by thrombocytopenia or leukopenia. The incidence of acute myeloid leukemia (AML) is 13% by the age of 50 years. Solid tumors, particularly those of the head, neck, skin, gastrointestinal tract, and genitourinary tract, are more common in individuals with FA. Molecular genetic testing is complicated by the presence of at least 15 genes that can be altered in the disease, many of which regulate DNA damage and repair pathways: BRCA2, BRIP1, PALB2, RAD51C, and SLX4 [57].Although various consequences in hematopoietic stem/progenitor cells have been attributed to FA-BMF, the quest to identify the initial pathological event is ongoing. To address this issue, Nakahata, Saito and other colleges established iPSCs from fibroblasts of six patients with FA and *FANCA* mutations. An improved reprogramming method yielded iPSC-like colonies from all patients, and iPSC clones were propagated from two patients. Quantitative evaluation of the differentiation ability demonstrated that the differentiation propensity toward the hematopoietic and endothelial lineages is already defective in early hemoangiogenic progenitors [58]. Expression levels of critical transcription factors were significantly downregulated in these progenitors. These data indicate that the hematopoietic phenotypes of FA patients arise from the HSCs, underlying the potential usefulness of iPSCs technology in elucidating the pathogenesis of FA-BMF.

#### 5.1.2. β-Thalassemia

Thalassemias are a group of diseases characterized by abnormal hemoglobin production. Underlying genetic causes are from mutations or deletions coding for alpha (α-thalassemia) or beta (β-thalassemia) hemoglobin. Recently, in a case demonstrating principle to application of iPSCs and genetic engineering, Luo, Sun and colleagues were able to generate beta-thalassemia (β-Thal) patient-specific iPSCs and mediate correction of the β-globin gene through the CRISPR/Cas9 technology [59]. Gene-corrected cells exhibited normal karyotypes and full pluripotency with no off-targeting effects. In terms of differentiation efficiency, gene-corrected β-Thal iPSCs showed increased embryoid body ratio and percentages of hematopoietic progenitor cells. More importantly, gene-corrected β-Thal iPSCs showed restored β-globin expression and reduced reactive oxygen species production compared with the parental line. This approach suggested hematopoietic differentiation efficiency of β-Thal iPSCs can be greatly improved once the causative genetic lesion is corrected, laying the groundwork for using gene-corrected, iPSCs-derived HSCs in a clinical setting.

### 5.2. Generation of iPSCs from Hematologic Malignancy

Recent progress in blood cell reprogramming has increased the feasibility of developing iPSC models for studying acquired blood diseases such as myeloproliferative neoplasms (MPNs), aplastic anemia, myelodysplastic syndrome (MDS), paroxysmal nocturnal hemoglobinuria (PNH) and other forms of leukemia. Given that the majority of disease-relevant mutations are restricted to hematopoietic lineages, the traditional iPSC generation from fibroblasts may lack the genetic information implicated in the disease development. Nevertheless, fibroblast-derived iPSCs can be still used for disease modeling by serving as germ line controls containing certain predisposing mutations or polymorphisms.

#### 5.2.1. Myeloproliferative Neoplasms

In characterizing a disease model for myeloproliferative neoplasms, Yeh and colleagues generated iPSC lines from CD34^+^ peripheral blood cells of two patients with JAK2-V617F defined myeloproliferative disorders (MPD), one evidencing polycythemia vera (PV) and the other with primary myelofibrosis (PMF). Mutations of the JAK2 kinase characterize more than 95% of PV patients and 50% of PMF patients, resulting in expanded erythropoiesis and myelopoiesis, respectively. The MPD-derived iPSCs containing the mutation appeared normal in phenotypes, karyotype, and pluripotency when maintained as undifferentiated cells [52]. However, upon directed hematopoietic differentiation, progenitor cells showed increased proliferation rates and skewed tendency to form erythroid colonies, similar to primary CD34+ cells of the parental PV cells from whom the iPSCs were derived [52]. 

iPSCs have been obtained from Chronic Myelogenous Leukemia (CML) [54,60]. CML is a myeloproliferative disease characterized by the presence of the Philadelphia chromosome, involving a translocation between chromosomes 9 and 22 producing the bcr-abl fusion protein and exhibiting constitutive tyrosine kinase signaling activity. CML originated from HSCs transformed by the BCR-ABL fusion gene resulted in elevated downstream tyrosine kinase signaling. While the CML and CML-derived iPSCs both expressed BCR-ABL, the CML-iPSCs were resistant to imatinib in contrast to their imatinib-sensitive CML parental line [60]. Differentiation of CML-iPSCs to immature progenitor cells (CD34+CD38−CD90+CD45+) also displayed imatinib resistance, while more differentiated cells (CD34−CD45+) recovered sensitivity. Interestingly, pathways critical for BCR-ABL fusion gene signaling, e.g., Ras/MAPK/ERK, PI3K/Akt, and JAK/STAT were still active in derived iPSCs. And although treatment with the tyrosine kinase inhibitor Imatinib (IM, first line treatment for CML) had no effect on the phosphorylation level of ERK, AKT, or JNK in CML-derived iPSCs, the phosphorylation of signal transducer and activator of transcription (STAT) 5 and V-srk avian sarcoma virus CT10 oncogene homolog-like (CRKL), often activated in CML cells, were significantly reduced. This discrepancy could be ascribed to some intrinsic mechanisms leading to activation of ERK, AKT, or JNK independently from BCR-ABL presence that is essential for iPSCs maintenance. 

Notably, studies by Amabile et al. used iPSC technology to examine the role of epigenetic changes in CML disease progression. By deriving Leukemia-iPSCs (LiPSCs) genetically matched but epigenetically distinct from the parental cell lines, the authors showed that: (1) genetic and epigenetic alterations are both required to maintain the leukemic potential; (2) BCR-ABL fusion protein is able to trigger DNA methylation changes that contribute to leukemia formation and (3) nuclear reprogramming can erase aberrant DNA methylation, thereby delaying the onset of the malignancy. This study and others [53] suggest that, in addition to the disease-derived iPSCs’ therapeutic potential, the reprogramming process per se could find a broad application as a research tool to dissect molecular mechanisms behind diseases’ pathogenesis. 

#### 5.2.2. Myelodysplastic Syndrome

Myelodysplastic syndromes (MDS) are clonal hematologic disorders characterized by ineffective hematopoiesis and a propensity to progression to AML. Somatic loss of one copy of the long arm of chromosome 7 (del(7q)) is a characteristic cytogenetic abnormality in MDS, well-recognized as a marker of unfavorable prognosis [61]. Kotini and colleagues derived del(7q) and isogenic karyotypically normal iPSCs from MDS patients and demonstrated that del(7q) iPSCs recapitulated disease-associated phenotypes, including impaired hematopoietic differentiation. These disease phenotypes can be reproduced in karyotypically normal cells by inducing hemizygosity of defined segments, specifically the 40 Mb region (7 q31.3–q31.6) on the long arm of chromosome 7 that impairs normal differentiation. The authors then compared gene expression between isogenic iPSCs harboring one or two copies of chromosome 7q by constructing del(7q) ORF library with 75 ORFs encoding 62 genes significantly reduced in del(7q) clones. They demonstrated that four genes, *HIPK2*, *ATP6V0E2*, *LUC7L2*, and *EZH2*, are able to partially rescue hematopoietic defects in del(7q) derived iPSCs [62].They also observed that hematopoietic differentiation and colony formation potential of two *EZH2* haploinsufficient clones established from human ESCs using a CRISPR/Cas9-based strategy substantially dropped at levels intermediate between those of normal and del(7q) derived iPSCs [62].

## 6. Conclusions

The boost of multiple technologies for iPSC generation during recent years has expanded the feasibility of utilizing iPSCs not only as a research tool but also as an attractive alternative therapeutic approach. The advent of several strategies of genome editing, combined with the possibility of generating patient-specific pluripotent stem cells, is transforming our understanding of the molecular mechanisms underlying genetic diseases and cancers [63]. In particular, great efforts have been directed to the hematology field, leading to significant advances and offering new possibilities for disease modeling, drug development and autologous cell therapy. 

The growing engagement of pharmaceutical companies in developing iPSC strategies is certainly contributing to the translational application of iPSC-technology from the bench-side to the bedside. Indeed, the global market for iPSCs is expected to reach $2.9 billion in 2018, with an annual growth rate of 19.7% for the five-year period from 2013 to 2018 (Induced Pluripotent Stem Cells: Global Markets, Report #BIO135A; www.bccresearch.com), resulting from the increasing research and development for chronic diseases and huge funding allocated by the government for drug discovery. 

Thus, the greatest challenges for the future in the iPSC field are not scientific but financial. Indeed, strong support from pharmaceutical industries and governments will be needed to move forward with iPSC-based therapies [64]. 

For drug discovery and disease modeling, researchers must be persistent and patient. “iPSCs can only shorten the discovery process, not skip it. There’s no magic. With iPSCs or any new technology, it still takes a long time” [64].

## Figures and Tables

**Table 1:** Stem cells in hematologic applications. Use of embryonic stem cells, hematopoietic stem cells, and induced pluripotent stem cells have their respective advantages and difficulties. Concerns arising include availability and source of material, ethical considerations, and transplantation barriers.

	Embryonic Stem Cells (ESCs)	Hematopoietic Stem Cells (HSCs)	Induced Pluripotent Stem Cells (iPSCs)
Source	Inner mass cells of blastocyst	Bone marrow donations; umbilical cord blood	Any somatic cell
Application	Basic science research; limited clinical application currently	Hematopoietic stem cell transplantation	Basic science research
Markers	SOX2, NANOG, Oct-4, SSEA-1, SSEA-3, SSEA-4 TRA-1-60, TRA-1-81 Frizzled5	CD34+, c-Kit−/low, Lin-, CD38-,Flt-3/Flk-2	Reactivation of embryonic stem cell markers, e.g., SOX2, NANOG, OCT-4, KLF4, SSEA-4, TRA-1-60
Derivation	Isolation from in vitro fertilized embryos	Purification fromdonations	Ectopic expression of ESC transcription factors: OCT3/4, SOX2, KLF4, c-MYC
Pros	Able to generate all three germ layers; Amenable to cell culture expansion while maintaining pluripotency	Not controversial; Can be harvested from bone marrow blood, can be mobilized to peripheral blood upon granulocyte-colony stimulating factor (G-CSF) induction, or obtained from umbilical cord blood donations; Rise in biobanking of umbilical cord blood increases amount of source material	Non-invasive isolation; Avoids Human Leukocyte Antigen Loci (HLA)-compatibility issues Can be genetically altered before transfusion; Expands available research areas; Recapitulates patient genome; Theoretically unlimited source material
Cons	Ethical concerns of using embryonic-derived cells; Limited source material	Restricted lineage differentiation; Dependent on HLA compatibility; Transplantation-associated risks: immune suppression, graft rejection, graft vs. host disease	Low efficiency of reprogramming;Incomplete programming, or “epigenetic memory”; No standardized protocol for production; Genetically unstable; Safety concerns; Maintenance of germline mutations; Insertional mutagenesis for integrating vectors

**Table 2:** Selection stages for generation of iPSCs.

	Induced Pluripotent Stem Cells (iPSCs) Protocol	Consideration
1. Choice of Cell Type	Adult mouse and human fibroblasts were used in first iPSCs experiments.	While iPSCs can in principle be generated from any somatic cell, in practice, there seems to be an inverse relationship between degree of differentiation and ease of reprogramming. Additionally, there is expanding concern for “memory” of the original cell, hindering the re-differentiation process downstream.
2. Dedifferentiation	Retroviral- or lentiviral-mediated expression of four pluripotent-specific genes: OCT3/4, SOX2, KLF4, and c-MYC (OSKM).	Concern for the transforming potential of c-MYC led to the identification of other factor substitutes. c-MYC was later deemed dispensible, and other factor combinations (Nanog, Lin28) have successfully generated iPSCs.Methods of delivery must also consider the effects of insertional mutagenesis when using integrating vectors. Non-integrating viruses, small-molecules, RNA- and transposon-based technologies are also currently being explored.
3. Selection	Transduced cells are cultured in embryonic stem cell (ESC) medium + antibiotics for 2–4 weeks with an ESC-specific marker, Fbx15, driving antibiotic resistance. Only reprogrammed cells can survive the selection process.	Although Fbx15 is expressed only in ESCs, it is not essential to ESC development and explains the partial reprogramming observed initially. Currently, Nanog-driven selection is favored instead.
4. Differentiation	Cultured with feeder cells and cytokines directing lineage-specific differentiation.	iPSCs can differentiate through (direct) addition of lineage-specific transcription factors or (indirect) culture in lineage-specific cytokines and growth factors. Protocols vary among laboratories.
5. Functional Testing	Expression of lineage-specific markers measured through PCR or immunofluorescence.	Functional tests are not standardized. Definition of lineage-specific characteristics vary among laboratories.
